# Frontline management of multiple myeloma patients: optimizing treatment for patients in the Gulf region

**DOI:** 10.46989/001c.128113

**Published:** 2025-02-11

**Authors:** Mahmoud Marashi, Khalil Al Farsi, Hussni Al Hateeti, Ahmad Alhuraiji, Hesham Elsabah, Honar Cherif, Anas Hamad, Kayane Mheidly, Hani Osman, Mohamad Mohty

**Affiliations:** 1 Department of Hematology Mediclinic City Hospital and Dubai Hospital; 2 Department of Hematology Sultan Qaboos University Hospital; 3 Department of Medicine, Division of Hematology Sheikh Shakhbout Medical City; 4 Department of Hematology Kuwait Cancer Control Center; 5 Department of Hematology and Bone Marrow Transplantation National Center for Cancer Care and Research; 6 Pharmacy Department National Center for Cancer Care and Research; 7 Department of Hematology Tawam Hospital; 8 Department of Clinical Hematology and Cellular Therapy Sorbonne Université

**Keywords:** Multiple myeloma, daratumumab, ASCT, frontline, Gulf, high-risk

## Abstract

Treatment options for newly diagnosed multiple myeloma (NDMM) have expanded dramatically over the last two decades, resulting in remarkable improvements in response rates and median survival times. In eligible patients, autologous stem cell transplant plays the central role of an overall treatment strategy comprising induction, transplantation, consolidation, and maintenance. In this article, we draw from our own collective clinical experience of treating patients with NDMM in the Gulf region to discuss treatment strategies in both transplant-eligible and -ineligible patients, as well as in high-risk patients. We present position statements for these distinct patient populations specifically for treatment in the Gulf region, where patients with NDMM have a younger median age than and different comorbidity profile from Western populations. We discuss how the introduction of anti-CD38 agents, including daratumumab and isatuximab, have had a major impact on the frontline treatment landscape in MM, with daratumumab-based quadruplet and triplet regimens emerging as the new standard of care in transplant-eligible and -ineligible patients, respectively. In addition, we advocate aggressive quadruplet treatment of high-risk patients with NDMM, as part of a strategy including single or tandem transplant when eligible. Finally, we discuss the clinical and practical rationale behind our statements, which is intended to serve as a useful reference for hematologists treating physicians within the Gulf region and beyond.

## 1. Introduction

Multiple myeloma (MM) is a plasma cell malignancy that accounts for 1–2% of all cancers, and is the third most common hematological malignancy worldwide.^1–3^ The disease is characterized by excessive clonal proliferation of abnormal plasma cells in the bone marrow, which ultimately leads to organ failure.^4^ Globally, there are ~188,000 incident cases and ~121,000 deaths from MM each year,^5^ with regional and ethnic differences, including an upward trend in cases in the Middle East and Africa.^6^ In particular, the United Arab Emirates (UAE) and Qatar are the two countries with the highest worldwide increases in MM incidence and mortality over the last 30 years.^7^ MM is usually a disease of older individuals, with most patients diagnosed between the ages of 60 and 70 years,^4^ although ~10% of cases occur in patients younger than 50 years of age.^8^ Newly diagnosed MM (NDMM) patients in the Gulf region tend to be younger than those in Western countries,^6^ in keeping with the young median age in Gulf countries relative to other developed countries,^9^ with one retrospective study of a tertiary care center in the UAE reporting a median age as young as 43 years.^10^

MM usually proceeds from an asymptomatic monoclonal gammopathy of undetermined significance (MGUS) stage, which progresses through a more advanced ‘smoldering’ asymptomatic stage into active MM.^1,6,11^ The classic features of MM presentation are elevated calcium, renal failure, anemia, and bone lesions (collectively referred to as ‘CRAB’ criteria), and diagnosis is normally made when there is end-organ damage attributable to these features, alongside clonal plasma cells comprising at least 10% of the bone marrow.^3^ MM is still considered largely uncurable, but in recent years advances in the treatment armamentarium have allowed substantial improvements to be made with regard to quality of life, symptom control, and prolongation of survival.^2,4,12^

In this article, we review current treatment strategies for frontline MM in transplant-eligible and -ineligible patients, as well as strategies for treating high-risk patients. We present position statements for these patient populations drawn from our own clinical experience of treating patients with MM in the Gulf region. In addition, we discuss the rationale behind our statements, which we believe can serve as a useful reference position for hematologists within the region and beyond.

## 2. Materials and Methods

A group of experts from across the Gulf region of the Middle East convened twice in June 2024. Those attendees who were able to, joined the meeting physically in Doha, Qatar, and others joined virtually. The discussion was moderated by one international independent expert from France (Dr M. Mohty). The expert group comprised nine hematologists and one pharmacist who were selected due to their recognized seniority and expertise in the management of MM. During these meetings, the expert group collectively discussed and agreed upon position statements for frontline treatment of MM in transplant-eligible, transplant-ineligible, and high-risk patients.

## 3. Frontline treatment of MM

### 3.1. Overview

Effective treatment options for NDMM have expanded rapidly over the last two decades, and there are a range of treatments classes available, with discrete mechanisms of action, including proteasome inhibitors (e.g., bortezomib, carfilzomib), immunomodulatory agents (e.g., thalidomide, lenalidomide), corticosteroids (e.g., dexamethasone, prednisolone), anti-CD38 monoclonal antibodies (e.g., daratumumab, isatuximab), as well as standard chemotherapies (e.g., doxorubicin, melphalan).^2,4^ As a result of these advances, median survival rates have almost tripled from 2.5 years before 1997 to more than 7 years today, representing a remarkable improvement.^4^ Standard of care (SOC) for eligible patients with NDMM is centered around autologous stem cell transplant (ASCT) following high-dose melphalan, and patients are initially assessed broadly as either transplant-eligible or transplant-ineligible.^3,13^ This initial risk stratification is based on a number of factors that determine likelihood of toxicity following treatment, and is ideally performed at a specialized transplant center.^4,13^ Patient age and presence/extent of comorbidities are typically the key criteria, but general fitness, performance status, and frailty assessments are often informative.^4^ While ASCT and high-dose melphalan are potentially life-threatening in older and/or frail individuals, age is by itself not prohibitive, as long as the patient has good general fitness.^4^

## 4. Transplant-eligible patients with NDMM

### 4.1. Review of key evidence

ASCT with high-dose melphalan has been the cornerstone of MM therapy for over two decades,^2^ and is used in eligible NDMM patients as part of an overall treatment strategy comprising induction, transplantation, consolidation, and maintenance therapy, an approach that is associated with excellent response and survival rates.^2^ Induction/consolidation with triplet therapy including a proteasome inhibitor, immunomodulatory drug, and dexamethasone, followed by maintenance with lenalidomide (R), has become a well-established standard of care in NDMM.^3,13^

The phase 3 IFM 2009 study showed that bortezomib, lenalidomide, and dexamethasone (VRd) with ASCT achieved a median progression-free survival (PFS) of 50 months and a complete response (CR) rate of 59% after 43-month median follow-up, with one-year fixed duration lenalidomide (R) maintenance.^14^ Building on these findings, the phase 3 DETERMINATION study later achieved a median PFS of 67.5 months and CR of 46.8% after 76-month median follow-up, using a similar study design to IFM 2009, but with continuous R maintenance until disease progression.^15^ Triplet therapy with bortezomib, thalidomide and dexamethasone (VTd) is another option for transplant-eligible patients with NDMM.^16–18^ However, in the absence of head-to-head trials, an integrated analysis has suggested VRd may have a better benefit–risk profile than VTd, including a higher very good partial response (VGPR) rate and less frequent adverse events leading to discontinuation.^19^

Introduction of the anti-CD38 monoclonal antibody daratumumab has had a major impact on the frontline treatment landscape of MM, and daratumumab-based quadruplets including Dara-VRd and Dara-VTd are emerging as the new SOC, with substantial improvements in efficacy outcomes observed, versus triplet therapy.^2,12^ The phase-2 GRIFFIN study investigated the addition of daratumumab to standard VRd (Dara-VRd) for 4 cycles of induction, ASCT, then 2 cycles of consolidation, followed by 26 cycles of maintenance with R±Dara.^20^ The study demonstrated that the addition of daratumumab to VRd improved the rate and depth of response compared with the VRd group, with no additional safety concerns. The findings of GRIFFIN have recently been confirmed and built upon in the phase 3 PERSEUS study, which investigated subcutaneous daratumumab (in contrast to the intravenous formulation used in GRIFFIN) in 709 transplant-eligible patients with NDMM.^20^ That study showed that after a median follow-up of 47.5 months, the risk of disease progression or death was significantly reduced with Dara-VRd, with a 48-month PFS rate of 84.3% versus 67.7% in the VRd group (hazard ratio [HR] 0.42; p<00001). Response rates were also superior with Dara-VRd, with 87.9% of patients achieving CR or better compared with 70.1% in the VRd group.^21^ Findings in PERSEUS were maintained across prespecified, clinically relevant subgroups, and support the use of Dara-VRd and daratumumab-R as SOC for transplant-eligible patients with NDMM. Of note, the incidence of peripheral neuropathy in PERSEUS was lower than historically reported, and this is likely due to increased clinical experience in adjusting doses of bortezomib, of which peripheral neuropathy is a known side effect.^22^

Daratumumab has also been investigated in the phase 3 CASSIOPEIA trial, as an addition to the VTd triplet (Dara-VTd).^23,24^ The latter was used in transplant-eligible patients as induction/consolidation, followed by daratumumab monotherapy maintenance, and was found to improve outcomes in NDMM patients, establishing Dara-VTd as an SOC in this setting. More recently, the phase 3 Iskia trial showed that adding the anti-CD38 monoclonal antibody isatuximab to a triplet of carfilzomib, lenalidomide and dexamethasome (Isa-KRd) resulted in significant clinical improvements compared with KRd alone, when used as consolidation/induction therapy in the transplant-eligible NDMM setting.^25^ However, carfilzomib is associated with increased cardiovascular adverse events,^26^ so restricting its use to high-risk patients may be preferable, in whom it has some clinical advantages (see later section). This may be especially prudent in the Gulf region, where rates of metabolic syndrome and risk for cardiovascular events are high.^27,28^

The key trials in frontline transplant-eligible patients and their primary outcomes are summarized in **Table 1**.

**Table 1.** Key trials in frontline transplant-eligible MM patients

Trial phase/name (Registration number)	Comparison / trial design	Patient N	Primary endpoint results	Primary reference
Phase 3 IFM 2009 (NCT01191060)	VRd ± ASCT; R maintenance for 1 year	700	PFS: median (VRd + ASCT *vs*. VRd) 50 months *vs.* 36 months; adjusted HR 0.65, p<0.001	*Attal et al., 2017^14^*
Phase 3 DETERMINATION (NCT01208662)	VRd ± ASCT; R maintenance (continuous)	722	PFS: median (VRd *vs*. VRd + ASCT) 46.2 months *vs.* 67.5 months; HR 1.53, p<0.001	*Richardson et al., 2022^15^*
Phase 2 GRIFFIN (NCT02874742)	Dara-RVd *vs.* RVd; R ± Dara maintenance	207	Stringent CR: (Dara-RVd *vs.* RVd) 42.4% *vs.* 32.0%; OR 1.57, 1-sided p=0.068 (prespecified 1-sided α=0.10)	*Voorhees et al., 2020^20^*
Phase 3 PERSEUS (NCT03710603)	Dara-VRd *vs.* VRd; R ± Dara maintenance	709	PFS: 48-month estimate (Dara-VRd *vs.* VRd) 84.3% *vs.* 67.7%; HR 0.42, p<0.001	*Sonneveld et al., 2024^21^*
Phase 3 CASSIOPEIA (NCT02541383)	Dara-VTd *vs.* VTd; Dara or observ. maintenance	1085	Stringent CR: (Dara-VTd *vs.* VTd) 29% *vs.* 20%; OR 1.60, p=0.0010	*Moreau et al., 2019^23^*
Phase 3 IsKIA (NCT04483739)	Isa-KRd *vs.* KRd; R maintenance per SOC	302	MRD negativity by NGS (10^-⁠5^): (Isa-KRd *vs.* KRd) 77% *vs.* 67%; OR 1.67, p=0.049	*Gay et al., 2023^25^*

Abbreviations: ASCT, autologous stem cell transplant; CR, complete response; d, dexamethasone; Dara, daratumumab; HR, hazard ratio; Isa, isatuximab; K, carfilzomib; Observ., observation; MRD, minimal residual disease; NGS, next-generation sequencing; PFS, progression-free survival; R, lenalidomide; SOC, standard of care; T, thalidomide; V, bortezomib; vs., versus

### 4.2. Position statement: frontline treatment for transplant-eligible patients

The following is our recommended position statement for SOC treatment in this setting:

Induction/consolidation: **Dara-VRd** (either 4 cycles as induction prior to transplant with 2 cycles after as consolidation, or 6 cycles as induction prior to transplant with no consolidation).

Maintenance: **R** (approved therapy) or **Dara-R** (select patients).

### 4.3. Expert clinical opinion

**Figure 1.**
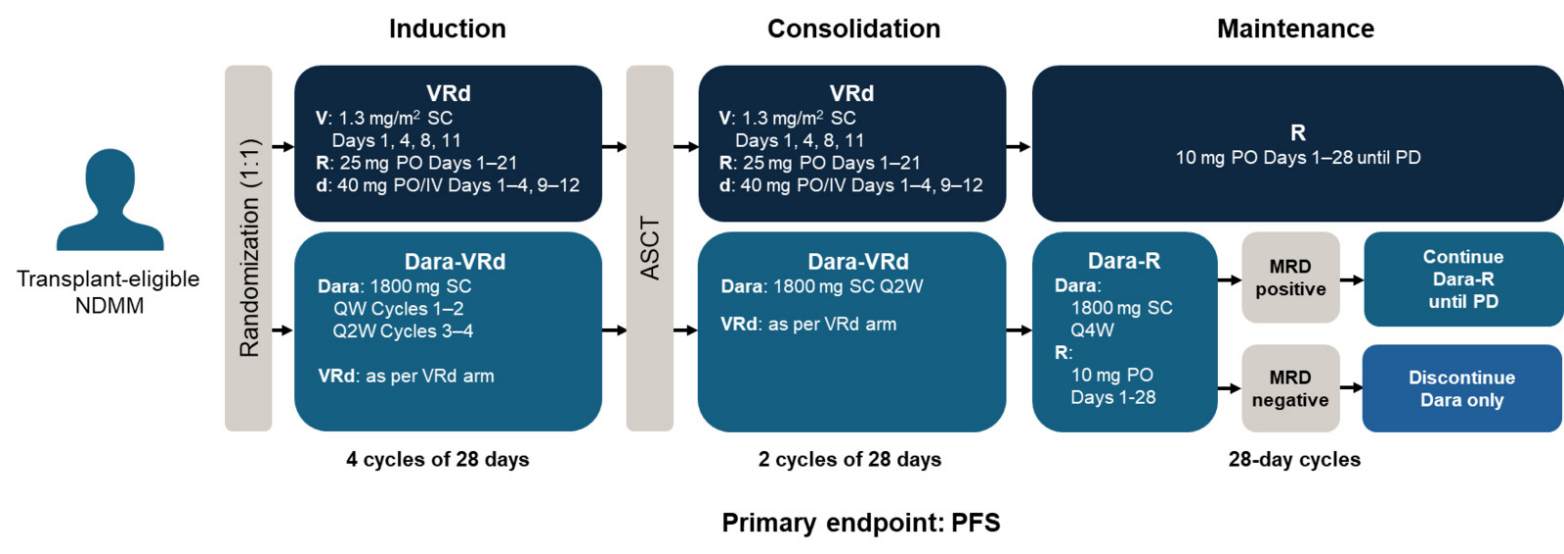
Study design of the phase 3 PERSEUS trial in transplant-eligible patients with NDMM Study design of the phase 3 PERSEUS trial.^21^ In our clinical practice, we have found that several standard adjustments can be made to the PERSEUS trial design to improve tolerability without compromising overall efficacy. Bortezomib can be administered once weekly instead of twice weekly to minimize risk of peripheral neuropathy. Lenalidomide 25 mg daily can be administered over 1–14 days, or at a reduced dose of 15 mg daily for 1–21 days, to avoid risk of neutropenia. Similarly, for dexamethasone, after the first two cycles, the dose can be reduced from 40 mg to 20 mg. Abbreviations: ASCT, autologous stem cell transplant; d, dexamethasone; Dara, daratumumab; IV, intravenous; MRD, minimal residual disease; NDMM, newly diagnosed multiple myeloma; PD, progressive disease; PFS, progression-free survival; PO, oral; QW, weekly; Q2W, every 2 weeks; Q4W, every 4 weeks; R, lenalidomide; SC, subcutaneous; V, bortezomib

We recommend using Dara-VRd as SOC for induction/consolidation in transplant-eligible NDMM patients in the Gulf region. This recommendation is based on the best available clinical evidence, primarily the phase-2 GRIFFIN trial and the phase-3 PERSEUS trial, as well as the relatively young median age of patients at diagnosis in the Gulf region who can tolerate quadruplet therapy well.^29^ The standard treatment protocol with Dara-VRd from the PERSEUS trial is shown in **Figure 1**, but it is common practice in our clinics to give bortezomib once weekly instead of the approved twice weekly dose, to minimize the risk of peripheral neuropathy. In addition, lenalidomide 25 mg daily is more commonly administered over 1–14 days, or at a reduced dose of 15 mg daily for 1–21 days, to avoid the risk of neutropenia. Similarly, for dexamethasone, the dose can be reduced from 40 mg to 20 mg after the first 2 cycles.

We routinely use quadruplet therapy, particularly in younger fit patients, and have achieved excellent stem cell mobilization and PFS results using this approach, with most patients still being in remission today. The tolerability of Dara-VRd is acceptable in younger patients, while additional dose modifications can be made in those aged ≥65 years to manage adverse events. These include reducing the dose of bortezomib or reducing its administrations, from once a week to once every two weeks, and switching to once monthly daratumumab rather than once every two weeks. Other options for managing adverse events include de-escalating treatment to Dara-Rd (i.e., removing bortezomib).

While VRd is still an excellent option in this patient population, using Dara-VRd has improved outcomes such that it has become SOC even for higher risk patients (see later section), and is preferable to VRd. In renal patients, Dara-VCd (replacing lenalidomide with cyclophosphamide) is a useful alternative where there are concerns about the adverse effect of lenalidomide on kidney function.^30^ If patients have an active infection, it is recommended to delay daratumumab initiation until the infection has cleared.

The PERSEUS and GRIFFIN trial designs followed 4 induction cycles and 2 post-ASCT consolidation cycles.^20,21^ Four induction cycles are preferred, but if this must be adjusted e.g., for logistical reasons, then it should not drastically affect outcomes as long as an overall 6 cycles of induction/consolidation are administered (minimum 2–3 cycles induction). In some cases, it can be difficult to bring patients back for consolidation due to post-transplant fatigue, so 6 cycles of induction can be an option, if this is a particular risk.

Regarding maintenance therapy, lenalidomide is the only approved therapy in NDMM, and is the preferred recommendation in current international guidelines.^3,13,31^ Recent data have shown that an MRD-driven duration of maintenance therapy is possible, even in patients with high-risk features.^32,33^ Thalidomide and bortezomib have also shown efficacy in the maintenance setting.^34^ The latter may have utility in patients with a lenalidomide allergy, and ongoing studies have also shown promise with iberdomide in the maintenance setting.^35,36^ However, the latest update from the phase-3 CASSIOPEIA trial with a median 80-month follow-up has shown that daratumumab monotherapy maintenance significantly improved PFS, compared with observation alone, after Dara-VTd induction/consolidation.^24^ Furthermore, the GRIFFIN and PERSEUS trials showed that Dara-VRd induction/consolidation followed by Dara-R maintenance was superior to VRd induction/consolidation and R maintenance,^20,21^ supporting the use of daratumumab across the entire treatment regimen. There is an argument that daratumumab could be excluded from maintenance treatment, thus reserving its use for later lines of therapy. Indeed, some patients have excellent outcomes on a lenalidomide fixed-duration maintenance strategy. However, there is no current way to predict which patients are likely to do well on the latter strategy. Therefore, due to attrition rates of around 50% following relapse, meaning that approximately half of patients never receive second-line treatment,^12,37^ we advocate giving the most effective therapy to achieve the maximum PFS in the frontline setting. If a patient has responded well to Dara-VRd and is likely to tolerate continuous Dara-R with a once monthly daratumumab subcutaneous injection, we would support proceeding with Dara-R maintenance.

As with any clinical management strategy, it is important to ensure there is an adequate supportive care plan in place to counter treatment- or disease-related adverse events in the NDMM setting. Good supportive care can have a major impact on patient quality of life, help prevent treatment delays or discontinuations and, therefore, ultimately has the potential to impact patient survival.^38^ Bone-directed therapies (e.g., bisphosphonates and RANK ligand inhibitors) can be used to counteract bone dysfunction due to lytic bone disease, which is common among patients with NDMM.^38^ Additional considerations for supportive care include intravenous immunoglobulins and anticoagulants, to combat risk of infection and adverse cardiac events, respectively, as well as agents that can counteract common gastrointestinal complaints, including diarrhea and constipation.^38^

## 5. Transplant-ineligible patients with NDMM

### 5.1. Review of key evidence

Patients with NDMM who are considered transplant-ineligible (e.g., due to older age, frailty, comorbidities) can be treated successfully with many of the same agents used as induction therapy prior to ASCT in transplant-eligible patients. However, double- or triple-agent regimens are generally preferred over quadruplet therapies, due to risks associated with frailty and older age in the transplant-ineligible population. The phase-3 SWOG S0777 trial demonstrated superiority of VRd to Rd in patients not immediately considered eligible for ASCT.^39^ After a median of 84-month follow-up, patients randomized to receive eight 21-day cycles of VRd achieved a median PFS of 41 months, compared with 29 months in those treated with six 28-day cycles of Rd, with both groups receiving a median of 17.1 months Rd maintenance therapy.^40^ In that study, the CR rate was 24.2% versus 12.1% in the VRd versus Rd groups, while VGPR rates were 74.9% versus 53.2%, respectively. However, in older patients aged ≥65 years, the same clinical benefits were not observed.^39,40^ As such, a dose-modified VRd protocol (‘VRd lite’) has been developed that provided excellent survival rates in an elderly population (median age of 72 years at study entry), with a median PFS of 41.9 months and 5-year overall survival (OS) of 61.3% after a median follow-up of 61 months.^41,42^

Similar to the landscape in transplant-eligible patients with NDMM, the introduction of daratumumab to treatment regimens has substantially improved outcomes in the transplant-ineligible setting. The phase-3 MAIA study in transplant-ineligible patients aged ≥65 years with multiple comorbidities investigated continuous Dara-Rd until disease progression or unacceptable toxicity.^43^ After a median follow-up of 64.5 months in MAIA, the Dara-Rd group achieved superior median PFS to that of Rd alone (61.9 versus 34.4 months), with an overall response rate of 93% versus 82%, and estimated 60-month OS of 66.6% versus 53.6%, respectively.^44^ Improved clinical benefits of Dara-Rd were observed across all age groups examined, including older patients aged up to 75 years, and in those with a single high-risk cytogenetic marker, establishing Dara-Rd as a standard of care in transplant-ineligible patients with NDMM.^12^ In the absence of head-to-head trials comparing Dara-Rd with VRd in the transplant-ineligible frontline MM setting, the PEGASUS study performed an indirect comparison of outcomes in MAIA with those in patients treated with common SOC regimens in community-based oncology centers.^45^ That study estimated that treatment with Dara-Rd was associated with significantly lower risk of death or progression compared with Rd (HR 0.54), VRd (HR 0.68), and Vd (HR 0.48). Of note, the ongoing phase-3 CEPHEUS trial is addressing whether adding Dara to VRd could further improve outcomes in transplant-ineligible patients.^46,47^ Initial results from CEPHEUS after a median follow-up of 58.7 months showed that the primary endpoint of overall MRD-negativity rate was higher in the Dara-VRd versus VRd arms (60.9% versus 39.4%), suggesting the daratumumab-based quadruplet regime improves outcomes compared with VRd.^48^

Daratumumab has also been examined in transplant-ineligible NDMM patients in combination with bortezomib, melphalan and prednisolone (Dara-VMP) in the phase 3 ALCYONE study.^49,50^ The study showed that after a median follow-up of 74.7 months, Dara-VMP achieved a median OS of 82.7 versus. 53.6 months in the VMP group, with benefits observed across the majority of subgroups examined.^51^ However, while Dara-VMP has become SOC and is listed in international guidelines,^3,13^ its use is not widespread, as melphalan-based regimens are often avoided due to concerns about genotoxicity.^52,53^ An alternative to VMP is bortezomib, cyclophosphamide and dexamethasone (VCD), and a recent randomized trial in transplant-ineligible frontline MM patients has shown that the addition of daratumumab to VCD (Dara-VCD) improved the median PFS versus VCD alone (25.8 versus 16.8 months), although this fell outside of statistical significance.^52^

Results from the phase-3 IMROZ trial have recently been reported,^54^ in which isatuximab plus VRd (Isa-VRd) was compared with VRd alone in frontline transplant-ineligible MM patients who were older but still fit. Isa-VRd reduced risk of disease progression or death by 40.4% (median PFS not reached versus 54.3 months with VRd), and the quadruplet was associated with deep and sustained responses (CR rate 74.7% versus 64.1%), leading to its recent approval,^55^ and supporting its use as a potential new SOC in this setting. In IMROZ, patients who experienced progressive disease in the VRd group were permitted to crossover to Isa-Rd continuous treatment, to offer them the best available treatment.^54^

The key trials in frontline transplant-ineligible patients and their primary outcomes are summarized in **Table 2**.

**Table 2.** Key trials in frontline transplant-ineligible MM patients

Trial phase/name (Registration number)	Comparison / trial design	Patient N	Primary endpoint results	Primary reference
Phase 3 SWOG S0777 (NCT00644228)	VRd *vs.* Rd	525	PFS: median (VRd *vs.* Rd) 43 months *vs.* 30 months; stratified HR 0.712, 1-sided p=0.0018	*Durie et al., 2017^39^*
Phase 3 MAIA (NCT02252172)	Dara-Rd *vs.* Rd	737	PFS: 30-month estimate (Dara-Rd *vs.* Rd) 70.6% *vs.* 55.6%; HR 0.56, p<0.001	*Facon et al., 2019^43^*
Phase 3 ALCYONE (NCT02195479)	Dara-VMP *vs.* VMP	706	PFS: 18-month estimate (Dara-VMP *vs.* VMP) 71.6% *vs.* 50.2%; HR 0.50, p<0.001	*Mateos et al., 2018^49^*
Phase 2 AMaRC 03-16 (ACTRN12617000202369)	Dara-VCD *vs.* VCD	121	PFS: median (VCD *vs.* Dara-VCD) 16.8 months *vs.* 25.8 months; HR 0.67, p=0.066	*Mollee et al., 2024^52^*
Phase 3 IMROZ (NCT03319667)	Isa-VRd *vs.* VRd	446	PFS: median (Isa-VRd *vs.* VRd) NR *vs.* 54.3 months; HR 0.596, log-rank p=0.0005	*Facon et al., 2024^54^*
Phase 3 CEPHEUS (NCT03652064)	Dara-VRd *vs.* VRD	395	Overall MRD-negativity rate (Dara-VRd *vs.* VRD) 60.9% *vs.* 39.4%; OR 2.37, p<0.0001	*Usmani et al 2024^48^*

Abbreviations: C, cyclophosphamide; d, dexamethasone; Dara, daratumumab; HR, hazard ratio; Isa, isatuximab; M, melphalan; OR, odds ratio; P, prednisone; PFS, progression-free survival; R, lenalidomide; V, bortezomib; vs., versus

### 5.2. Position statement: frontline treatment for transplant-ineligible patients

The following is our recommended position statement for standard-of-care treatment in this setting:

All patients (except category below)

Treatment: **Dara-Rd** – until disease progression.

Fit patients aged 65–70 years (borderline transplant-eligible)

Treatment: **Dara-Rd** (6–8 cycles). Dara-R to be continued until disease progression.

Note: Patients who received **Dara-Rd** may be able to progress to transplant and should be considered in specific circumstances, for example, in fit patients who are borderline transplant-eligible, or for whom there is a change in patient/physician decision to proceed (usually between age 65 and 70 in the Gulf region).

### 5.3. Expert clinical opinion

**Figure 2.**
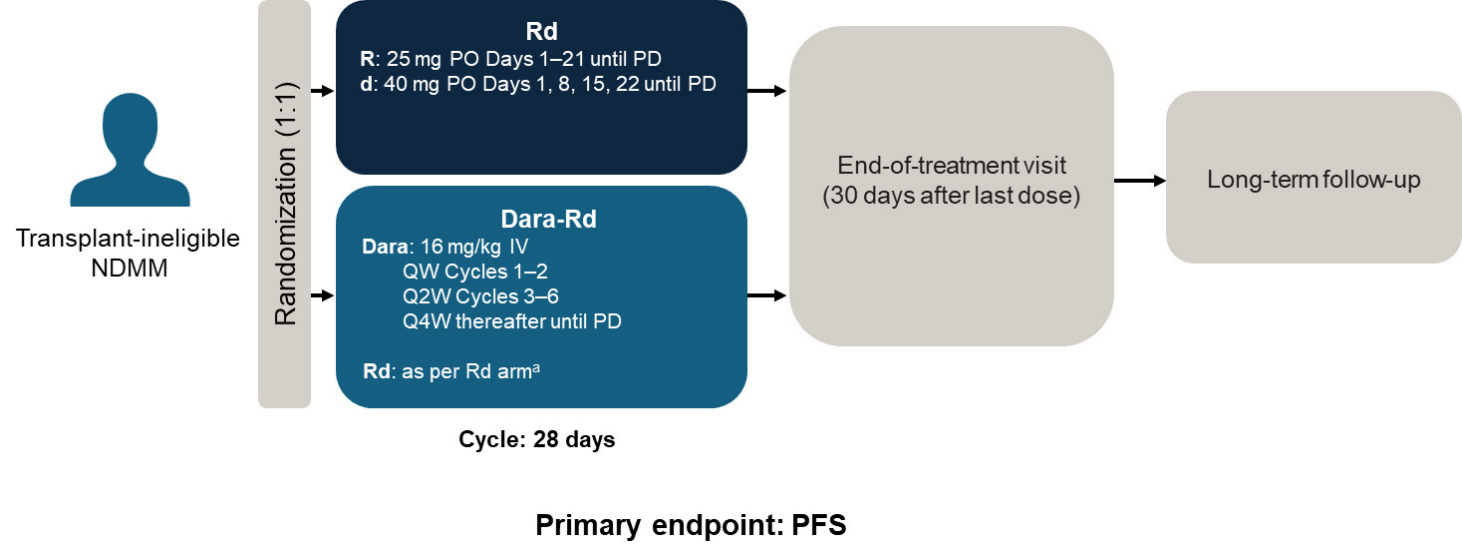
Study design of the phase 3 MAIA trial in transplant-ineligible patients with NDMM Study design of the phase 3 MAIA trial.^43^ ^a^On days when daratumumab was administered, dexamethasone was administered by IV to serve as the daily steroid treatment dose and the pre-infusion medication. Abbreviations: d, dexamethasone; Dara, daratumumab; IV, intravenous; NDMM, newly diagnosed multiple myeloma; PD, progressive disease; PFS, progression-free survival; PO, oral; R, lenalidomide; QW, weekly; Q2W, every 2 weeks; Q4W, every 4 weeks

There are currently two main SOC treatments used in frontline transplant-ineligible patients, VRd and Dara-Rd. Although Dara-VMP is recommended,^3,13^ it is not widely used in this setting, due to concerns with extended use of melphalan-based therapies. Of the available treatments, we believe that continuous Dara-Rd, with a reported median PFS of 62 months in the MAIA trial^43^ (study design in **Figure 2**), is the most suitable for patients in the Gulf region. Having a once monthly hospital visit for a daratumumab injection is not necessarily an inconvenience for elderly patients and, in fact, many patients appreciate the opportunity to have a regular appointment where they are observed in a clinical setting, and are potentially able to see other specialists for comorbidities. Data from the MAIA trial showed that health-related quality of life improved with Dara-Rd, where it was associated with improvements in physical functioning and a notable reduction in pain from baseline throughout therapy duration.^56^

In our clinical experience, Dara-Rd is very well tolerated even in patients aged over 85 years. If necessary, it is possible to improve tolerance to the regimen by reducing the doses of lenalidomide to ≤15 mg and dexamethasone to ≤10 mg. An ongoing study is investigating a dexamethasone-sparing strategy in frail patients with NDMM, and has shown favorable results with a Dara-R regimen compared with Rd alone, suggesting Dara-R may be an alternative option for initial treatment in selected transplant-ineligible patients in the future.^57,58^ For frail patients who are bed-ridden, infection-prone, or in those who are not willing to travel to hospital, oral treatment with Rd would be the only option. Dara-Vd is a good option in renal patients, possibly switching to Dara-Rd after 1–2 cycles.

The results of the IMROZ trial showed that adding isatuximab to VRd improves outcomes in transplant-ineligible patients, which led to the approval of Isa-VRD in this setting,^55^ while recent results of the CEPHEUS trial have shown that Dara-VRd is also of benefit in this setting.^48^ In principle, Dara-VRd may be preferable to Isa-RVd, due to its subcutaneous administration and avoidance of infusion-related reactions, as well as a lack of experience with isatuximab among physicians in the Gulf region. However, in our personal experience, Dara-VRd should only be used in younger patients (65–70 years) who are fit and healthy, and it can be used successfully to avoid ASCT. Quadruplet therapy should generally be avoided in very old or frail patients. Additionally, elderly patients who struggle with frequent hospital visits may benefit from treatments that include the oral proteasome inhibitor ixazomib. Phase-2 studies with the combination of ixazomib, lenalidomide, and dexamethasone (IRd) or Dara-Id support this approach.^59,60^ Of note, the age at which patients (even if fit) are considered too old for ASCT can vary by country and local practice. For example, in some Gulf countries, ASCT can be considered too risky in patients over the age of 65 years.

## 6. High-risk patients with NDMM

### 6.1. Review of key evidence

Despite tremendous improvements in outcomes in NDMM since the introduction of new treatment classes, there is still a subset of patients that does not respond well to standard treatment approaches. Such high-risk patients are characterized by treatment resistance, minimal residual disease (MRD) positivity, relapse, early progression, and death.^61,62^ Historically, patients at high risk of relapse in MM were primarily defined based on the presence of cytogenetic abnormalities, including chromosomal translocations (particularly t[4;14], t[14;16], and t[14;20]), deletions (e.g., del[17p], del[1p]), and amplifications (e.g., amp1q).^61,62^ However, it is generally accepted that classifying high-risk patients should also take into account additional disease-related factors (e.g., extramedullary disease, disease aggressiveness, biochemical changes) as well as patient-related factors (e.g., age, frailty, comorbidities).^13,63^

The choice of therapy for high-risk patients will vary depending on eligibility for ASCT. Several treatment strategies have been explored in the high-risk setting, including varying the choice/aggressiveness of frontline therapy, considering use of tandem ASCT (if eligible), increasing treatment consolidation length, and proceeding with a continuous multidrug maintenance therapy. A systematic review and meta-analysis including patients with NDMM and high-risk features from the CASSIOPEIA, MAIA, and ALCYONE trials, reported that the addition of daratumumab to various backbone regimens was associated with improved PFS,^64^ suggesting the clinical benefits of daratumumab also apply in the high-risk setting. A separate pooled analysis suggested this was also true when restricted to transplant-ineligible patients only (MAIA and ALCYONE).^65^

In the transplant-eligible setting, a post-hoc analysis of NDMM patients with high-risk cytogenetic anomalies (HRCAs) from the GRIFFIN trial showed higher MRD-negativity rates in the Dara-VRd versus VRd arm (54.8% versus 32.4%).^66^ In addition, rates were better in patients aged ≥65 years (67.9% versus 17.9%, respectively) and in those specifically with 1q21 gains or amplifications (61.8% versus 28.6%, respectively), which are among the most common HRCAs observed in NDMM.^67^ These results are in keeping with the phase-3 PERSEUS trial, where the Dara-VRd group had better responses than the VRd group in a subgroup analysis of patients with high cytogenetic risk.^21^ Daratumumab-based regimens have been further examined in the phase-2 single-arm MASTER trial, which investigated Dara-KRd and ASCT in a population predominantly of patients with HRCAs, using a proportion of patients reaching MRD-negativity at any time during therapy as a primary endpoint.^68^ In this study’s final analysis, the 36-month PFS was 88% among patients with no HRCAs, 79% for those with one HRCA, and 50% for those with two or more HRCAs, suggesting that while the quadruplet showed some success among high-risk patients, there is still substantial room for improvement in outcomes among those with ultra-high-risk disease (i.e., those with ≥2 HRCAs).^69^

Other approaches have been investigated in high-risk NDMM. The phase-2 OPTIMUM study looked at induction therapy with Dara-CVRd (where C is cyclophosphamide) prior to ASCT in ultra-high-risk patients, followed by an extended consolidation period of Dara-VRd for 6 cycles and an additional 12 cycles with Dara-VR, followed by Dara-R maintenance.^70^ That study reported a 30-month PFS rate of 77%, which was compared to a value of 39.8% calculated using a pre-specified digital comparison with patients in the MyeXI trial, which recruited ultra-high-risk patients treated with carfilzomib-cyclophosphamide-lenalidomide-dexamethasone (KCRd) or CRd induction. Another approach for treating high-risk patients is tandem ASCT, which has historically been controversial in NDMM, but recent studies have shown its potential benefit in this setting.^71,72^ The phase-2 IFM 2018-04 trial investigated a Dara-KRd-based tandem ASCT protocol in patients with high-risk NDMM.^73^ The trial design was 6 induction cycles of Dara-KRd prior to first ASCT, followed by consolidation with 4 cycles of Dara-KRd then second ASCT, and finally maintenance with Dara-R for 2 years.^73^ The efficacy was extremely encouraging, with a CR rate of 81% and MRD-negativity among evaluable patients of 94%; the 24-month PFS was 87% and 24-month OS was 94% after a median follow-up of 32 months. Although the study met the primary endpoint of feasibility of the intensive treatment strategy, whereby 72% of patients completed a second transplant, in practical terms, having nearly 30% of patients unable to tolerate the protocol, this may be prohibitively high.

Isatuximab-based regimens have also been investigated in transplant-eligible and non-eligible high-risk patients with NDMM in the phase 2 GMMG-CONCEPT trial.^74^ In transplant-eligible patients, induction was with Isa-KRd for 6 cycles prior to ASCT, then 4 consolidation cycles with Isa-KRd and maintenance with Isa-Kd for 26 cycles. Transplant-ineligible patients followed an identical protocol but with an additional 2 induction cycles instead of ASCT. The primary endpoint of MRD-negativity after consolidation was met with rates of 67.7% in transplant-eligible patients and 54.2% in transplant-ineligible patients, with a median PFS not reached in either arm after a median follow-up of 44 and 33 months, respectively.

The key trials in frontline high-risk patients and their primary outcomes are summarized in **Table 3**.

**Table 3.** Key trials in frontline high-risk MM patients

Trial phase/name (Registration number)	Comparison / trial design	Patient N	Primary endpoint results	Primary reference
Phase 2 MASTER trial (NCT03224507)	Dara-KRd + ASCT in patients with 0, 1, and 2+ HRCAs	123	MRD negativity by NGS (10^-5^): 80% overall; 78%, 82%, and 79% with 0, 1, and 2+ HRCAs, respectively	*Costa et al., 2022^68^*
Phase 2 OPTIMUM trial (NCT03188172)	Dara-CVRd* induction, ASCT, Dara-VR(d) consolidation, Dara-R maintenance	103	PFS: 18-month estimate 81.7% compared with 65.9% in a digital comparison of MyeXI trial patients treated with (K)CRd induction	*Kaiser et al., 2023^70^*
Phase 2 IFM 2018-04 (NCT03606577)	Dara-KRd^†^, tandem ASCT, Dara-R maintenance	50	Feasibility of intensive strategy: 72% patients completed second transplant (primary endpoint met)	*Touzeau et al., 2023^73^*
Phase 2 GMMG-CONCEPT (NCT03104842)	Transplant-eligible^‡^: Isa-KRd, ASCT; maintenance Isa-Kd	99	MRD negativity by NGS (10^-5^): 67.7%	*Leypoldt et al., 2024^74^*
	Transplant-ineligible^‡^: Isa-KRd; maintenance Isa-Kd	26	MRD negativity by NGS (10^-5^): 54.2%	

Abbreviations: ASCT, autologous stem cell transplant; CR, complete response; d, dexamethasone; Dara, daratumumab; HRCA, high-risk cytogenetic abnormality; HR, hazard ratio; Isa, isatuximab; K, carfilzomib; Observ., observation; MRD, minimal residual disease; NGS, next-generation sequencing; PFS, progression-free survival; R, lenalidomide; SOC, standard of care; T, thalidomide; V, bortezomib; vs., versus

*Ultra–high-risk patients defined as having ≥2 HRCAs;

†High-risk patients defined as the presence of del17p, t(4;14) and/or t(14;16);

‡High-risk patients defined as the presence of ≥1 of: del17p (in >10% of purified cells), t(4;14), t(14;16), or amp1q21

### 6.2. Position statement: frontline treatment for high-risk patients

The following is our recommended position statement for standard-of-care treatment in this setting:

Induction/consolidation: **Dara-VRd** and **ASCT** if feasible. **Dara-KRd** could be considered for selected patients without cardiovascular comorbidities.

Maintenance: **Dara-R**.

### 6.3. Expert clinical opinion

In general, the aggressive nature of the disease in high-risk patients warrants an aggressive treatment approach, and there are now specific protocols in place for them, including a second ASCT. However, treating high-risk patients can be complicated, and the presence of significant comorbidities such as renal or cardiac disease in themselves can limit treatment options, pushing patients into the high-risk category who would not otherwise be at elevated risk of poor outcomes.

**Figure 3.**
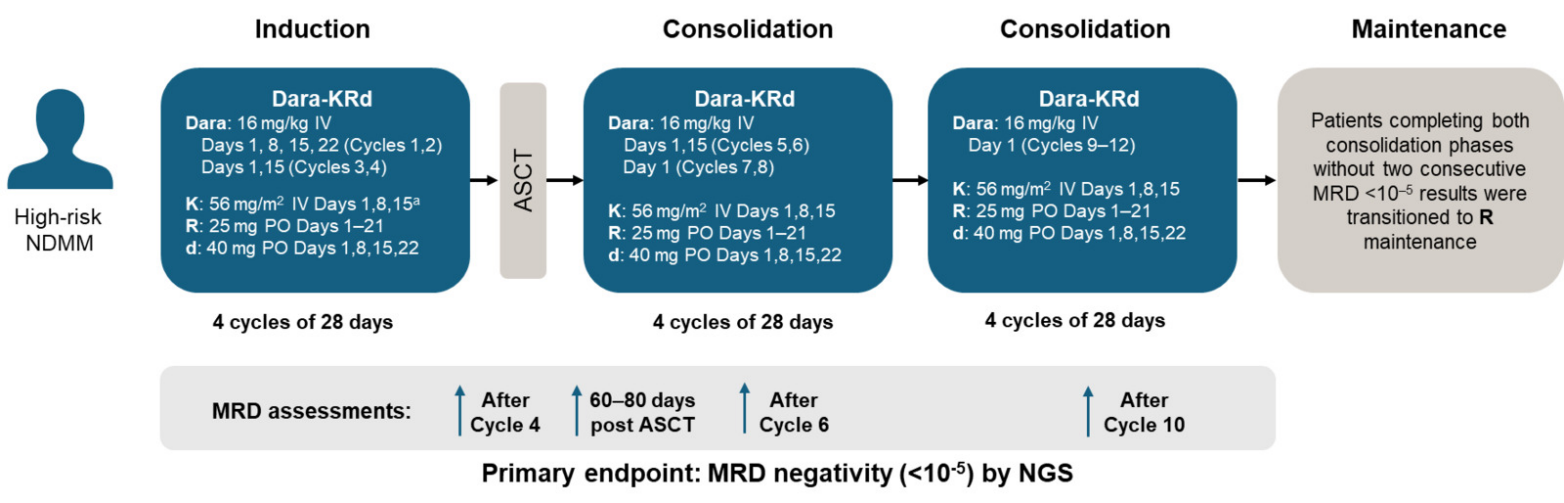
Study design of the phase-2 MASTER trial in high-risk patients with NDMM Study design of the phase-2 MASTER trial.^68^ ^a^20 mg/m^2^ on first dose of cycle Abbreviations: ASCT, autologous stem cell transplant; d, dexamethasone; Dara, daratumumab; IV, intravenous; K, carfilzomib; MRD, minimal residual disease; NDMM, newly diagnosed multiple myeloma; NGS, next-generation sequencing; PO, oral; R, lenalidomide

The goal of therapy in high-risk patients is to achieve a deep response and eradicate MRD inside and outside of the bone marrow, and we are in favor of using combinations of the most effective treatments upfront as continuous therapy. In general, we advocate Dara-VRd for upfront induction/consolidation treatment in the high-risk setting. Using carfilzomib instead of bortezomib could be considered in patients without cardiovascular disease, as was demonstrated in the phase-2 MASTER trial (**Figure 3**).^68^ However, due to the high incidence of cardiovascular risk factors and metabolic syndrome in the Gulf region,^27,28^ this would need to be done with caution. The addition of cyclophosphamide to Dara-VRd is also an option if a very aggressive approach is required, but it needs to be balanced against the likelihood of a reduced tolerability profile.

For maintenance, we recommend Dara-R as SOC for Gulf region patients. There is some evidence that adding bortezomib to maintenance may improve survival outcomes in high-risk patients^75^ however, this finding has recently been challenged,^76^ and the risk of peripheral neuropathy among populations with high levels of diabetes^77^ (as it is the case in the Gulf region) may offset any potential benefits. From a practical standpoint, finding a reliable and high-quality source for cytogenetic analyses (e.g., fluorescence in situ hybridization [FISH]) is essential, as around 25% of patients are expected to be at higher risk of HRCAs; a centralized laboratory serving the Gulf region would be ideal and, although there are logistical and administrative challenges with centralization, such an approach would have great benefits for the region.

Looking at the future, the emergence of chimeric antigen receptor (CAR) T-cell therapy looks highly promising regarding the treatment of frontline high-risk patients with MM. CAR-T cell therapy involves genetically modifying a patient’s own T-cells to recognize and target tumor antigens for destruction, with two having been already approved in the relapsed/refractory MM setting (idecabtagene vicleucel [ide-cel] and ciltacabtagene autoleucel [cilta-cel]) after showing impressive efficacy, including in high-risk, heavily pre-treated patients.^78,79^ Several initial studies suggest that CAR-T is feasible in the NDMM setting, and there are several ongoing, large, randomized trials to investigate the currently approved regimens in the frontline MM setting.^80^

## 6. Discussion and conclusion

The treatment landscape for NDMM has improved substantially over the last 20 years or so, with numerous classes of treatment with differing mechanisms of action now available. None has had a bigger impact than the anti-CD38 monoclonal antibodies, which have resulted in some of the best response and survival rates across transplant-eligible, -ineligible, and the high-risk settings. Looking to the future, CAR T-cell therapy holds promise in improving outcomes further in this currently incurable patient population, which will be particularly welcomed among the relatively young patient population in the Gulf region.

### Authors’ Contribution

Conceptualization: Mahmoud Marashi (Equal), Khalil Al Farsi (Equal), Hussni Al Hateeti (Equal), Ahmad Alhuraiji (Equal), Hesham Elsabah (Equal), Honar Cherif (Equal), Anas Hamad (Equal), Kayane Mheidly (Equal), Hani Osman (Equal), Mohamad Mohty (Equal). Data curation: Mahmoud Marashi (Equal), Khalil Al Farsi (Equal), Hussni Al Hateeti (Equal), Ahmad Alhuraiji (Equal), Hesham Elsabah (Equal), Honar Cherif (Equal), Anas Hamad (Equal), Kayane Mheidly (Equal), Hani Osman (Equal), Mohamad Mohty (Equal). Formal Analysis: Mahmoud Marashi (Equal), Khalil Al Farsi (Equal), Hussni Al Hateeti (Equal), Ahmad Alhuraiji (Equal), Hesham Elsabah (Equal), Honar Cherif (Equal), Anas Hamad (Equal), Kayane Mheidly (Equal), Hani Osman (Equal), Mohamad Mohty (Equal). Writing – original draft: Mahmoud Marashi (Equal), Khalil Al Farsi (Equal), Hussni Al Hateeti (Equal), Ahmad Alhuraiji (Equal), Hesham Elsabah (Equal), Honar Cherif (Equal), Anas Hamad (Equal), Kayane Mheidly (Equal), Hani Osman (Equal), Mohamad Mohty (Equal). Writing – review & editing: Mahmoud Marashi (Equal), Khalil Al Farsi (Equal), Hussni Al Hateeti (Equal), Ahmad Alhuraiji (Equal), Hesham Elsabah (Equal), Honar Cherif (Equal), Anas Hamad (Equal), Kayane Mheidly (Equal), Hani Osman (Equal), Mohamad Mohty (Equal).

### Competing of Interest – COPE

Mahmoud Marashi has nothing to declare

Khalil Al Farsi has nothing to declare

Hussni Al Hateeti has nothing to declare

Ahmad Alhuraiji has nothing to declare

Hesham Elsabah has nothing to declare

Honar Cherif has nothing to declare

Anas Hamad has nothing to declare

Kayane Mheidly has nothing to declare

Hani Osman has nothing to declare

Mohamad Mohty declares honoraria from Adaptive Biotechnologies, Amgen, Astellas Pharma, Bristol-Myers Squibb, GlaxoSmithKline, Janssen Cilag EMEA, Jazz Pharmaceuticals, Medac Pharma Inc., Novartis, OncoPep, Inc., Pfizer, Sanofi, Takeda Oncology, and Therakos.

### Ethical Conduct Approval – Helsinki – IACUC

(for more information, read https://chi.scholasticahq.com/for-authors)

Not applicable.

### Informed Consent Statement

Not applicable.

### Data Availability Statement

Not applicable.
